# Silicon Amendment Reduces Soil Cd Availability and Cd Uptake of Two *Pennisetum* Species

**DOI:** 10.3390/ijerph16091624

**Published:** 2019-05-09

**Authors:** Qiyu Dong, Jianbo Fang, Fei Huang, Kunzheng Cai

**Affiliations:** 1 Department of Ecology, College of Natural Resources and Environment, South China Agricultural University, Guangzhou 510642, China; holmesball@163.com (Q.D.); feihuang@scau.edu.cn (F.H.); 2 Key Laboratory of Tropical Agro-Environment, Ministry of Agriculture, South China Agricultural University, Guangzhou 510642, China; 3 College of Forestry and Landscape Architecture, South China Agricultural University, Guangzhou 510642, China; Jianbo2223@163.com

**Keywords:** toxic metal elements, soil, cadmium, silicon, *Pennisetum*

## Abstract

Silicon (Si) plays important roles in alleviating heavy metal stress, but the migrating effects and mechanisms, especially for *Pennisetum*, are not well studied. In this study, *Pennisetum glaucum* and *Pennisetum glaucum* × *P. purpureum* were used to explore the impacts of Si application on alleviating cadmium (Cd) toxicity and its possible mechanism. Treatments consist of four levels of Cd (0, 10, 50, and 100 mg·kg^−1^) with or without 2.0 mM Si amendments. Under Cd stress, Si application significantly increased plant biomass and Si content, reduced Cd content, and decreased the enrichment factor in shoots and roots. Si treatment also increased soil pH and soil residual Cd, while reducing available/oxidizable/reducible Cd content in soil at 50 and 100 mg·kg^−1^ Cd levels, thereby leading to a reduction of the soil’s available Cd. These findings indicate that Si application is effective in alleviating Cd phytotoxicity of *Pennisetum*, mainly through reducing plant Cd uptake and increasing soil pH and Cd immobilization, thereby reducing Cd bioavailability.

## 1. Introduction

Cadmium (Cd), one of the most common toxic elements, is mainly discharged from industrial activities, such as mining, refining, plastic manufacturing, and electroplating [1]. Due to its high mobility and assimilability, Cd can easily get into plants through root uptake and being translocated to shoots in an ionic form in xylem and phloem through transporters and transpiration [2,3,4,5], causing a series of toxic effects on plants, including leaf chlorosis, growth inhibition, mineral nutrient disorders, accumulation of reactive oxygen species, protein denaturation, and even plant death [6,7]. Moreover, Cd can enter human organs via the food chain, consequently posing severe risks to human health, such as kidney, bone, and pulmonary damage [6,7,8,9,10,11]. Thus, developing reliable approaches to prevent heavy metal accumulation in plants is vital for reducing the potential risk in edible parts of crops.

Silicon (Si), one of the most abundant elements in the earth’s crust, is second only to oxygen and accounts for 29.50% of the soil constituents [1,12]. Si has not been included as an essential element in higher plants [13], but many studies have shown that Si can be actively absorbed, translocated, and accumulated in massive amounts in many plants [14], and plays an important role in alleviating plant biotic and abiotic stresses [15,16]. It has been well documented that silicon enhances the tolerance of plants to toxic metal elements, including cadmium (Cd) [17], zinc (Zn) [18], manganese (Mn) [19], aluminum (Al) [20], chromium (Cr) [1], lead (Pb) [21], and copper (Cu) [22]. From previous research on crops, Si can alleviate the toxic effects of toxic metal elements on plants through external mechanisms and internal mechanisms. The external mechanisms include influencing pH and reducing the availability of toxic metal elements in soil, while internal mechanisms include influencing plant uptake, binding toxic metal elements in the cell walls, blocking the transport of toxic metal elements from roots to shoots, strengthening the defense system, and regulating protein and gene expression [23,24,25,26,27,28]. Farooq et al. [29] found that Si addition markedly enhanced cotton growth, gas-exchange parameters, and antioxidant enzyme activity in leaves, while decreasing Cd absorption, along with H_2_O_2_ and MDA content. Howladar et al. [30] found that silicon in different application methods increased polyamine contents and their gene expression. Vaculík et al. [31] showed that Si influenced the development of Casparian bands and suberin lamellae and vascular tissues in roots, leading to a decrease of Cd in symplasm in maize shoots. In the in situ analysis of cellular fluxes of the Cd^2+^ in suspension cells and root cells of rice exposed to Cd^2+^, Ma et al. [32] and Liu et al. [33] suggested that Cd ion uptake in rice could be suppressed by Si co-deposition of Si and Cd in the cell walls via a [Si-hemicellulose] Cd co-complexation; furthermore, Cd uptake in rice can be suppressed by Si. Molecular evidence showed that Si reduced Cd uptake of (1) wheat, by down-regulating LCT1 and HMA2 proteins and up-regulating PCS1 and IRT1 proteins [34], and (2) rice, by down-regulating the transporter genes, *OsNramp5* and *OsHMA2* [35].

*Pennisetum* is annual and perennial high-quality forage of Gramineae, which is widely cultivated in tropical and subtropical regions. It has large biomass, luxuriant foliage, extended branches, a well-developed root system, fast growth, and strong stress resistance [36]. *Pennisetum* is rich in nutrition and can be used as green fodder and as silage material for herbivorous poultry and herbivorous fish. It has the functions of soil and water conservation, wind and sand fixation, energy supply, and it can also be used as an ornamental plant. Furthermore, *Pennisetum* is highly tolerant to heavy metals, it has a strong accumulation capacity, and is used as an environmental remediation grass species [37]. At present, the mitigating effect of Si on toxic metal elements in plants mostly focuses on crops, but is not reported in grass so far. Therefore, this study was conducted to: (1) Investigate the impacts of exogenous Si application on plant growth, Cd uptake in *Pennisetum* plants, and soil Cd availability at different doses of Cd contamination; and (2) identify the possible mechanisms involved in Si-mediated Cd detoxification.

## 2. Materials and Methods

### 2.1. Plant Materials and Growth Conditions

*Pennisetum glaucum* and *Pennisetum glaucum* × *Pennisetum purpureum* were used as experimental materials. *P. glaucum* is an annual grass of Gramineae found in Africa. It has the characteristics of an erect-stem, strong-root system with corrugated-edge leaves. *P. glaucum × P. purpureum* is a hybrid of *P. glaucum* and *P. purpureum*, and it is a perennial grass of Gramineae. It has thick stems, better tillering ability, strong fibrous roots, and long broad leaves with bristles on the edges and thin hair on the surface. The *Pennisetum* seeds were provided by Henan Shijitianyuan Ecological Technology Limited Company, China.

### 2.2. Experimental Treatments

The experiment was performed in the greenhouse of the South China Agricultural University Ecological Farm (23.21 °N, 113.42 °E). Full and lustrous *Pennisetum* seeds were surface sterilized in 3% H_2_O_2_ for 20 min, and then rinsed with deionized water 3 times [1]. The seeds were germinated by immersion in deionized water at 28 °C in the dark for 24 h. After germination, six uniform seedlings were selected and transferred to plastic pots (22 cm × 26 cm × 17 cm), containing 2.5 kg soil. The soil was artificial soil mixed with peat soil and sand. The properties of the soil are shown in Table 1. To ensure the normal growth of *Pennisetum* during the experiment, urea (1.70 g·pot^−1^) and potassium dihydrogen phosphate (1.16 g·pot^−1^) were employed as base fertilizers in each pot before transplanting, and the fertilizer was supplemented according to the development during the growth period.

Eight treatments with three replicates were involved in this experiment: Four levels of Cd supply (0, 10, 50, and 100 mg·kg^−1^) with or without 2.0 mM Si application for *P. glaucum* and *P. glaucum × P. purpureum.* CdCl_2_ and K_2_SiO_3_ were mixed into the soil, and soil moisture was kept at 60–70% of the field water holding capacity for 7 days. After two months, plant and soil samples were collected for the determination of various parameters.

### 2.3. Plant Samples Analysis

Harvested plant samples in each treatment were separated into roots and shoots, dried at 110 °C for 30 min, and then dried at 65 °C until the materials reached their constant weights. The biomass of shoots and roots was weighed. Si concentration in roots and shoots was measured according to the modified autoclave-induced digestion method by Elliott et al. [38]. In brief, dried plant samples were dissolved with 3 mL of 50% NaOH solution at 121 °C for 2 h. Afterward, 30 mL 20% acetic acid, 10 mL ammonium molybdate (54 g·L^−1^, pH 7.0), 5 mL 20% tartaric acid, and 1 mL reductant were added to 1 mL of the samples. After 30 min, Si concentration of the samples was measured at 650 nm on a TU-1901 UV-Vis spectrophotometer (TU-1901, Beijing Purkinje General Instrument Co., Ltd., Beijing, China). To determine Cd concentration in plants, the dried plant samples (roots and shoots) were ground into powder and digested with a mixture solution (9 mL HNO_3_ and 1 mL H_2_O_2_), using a microwave digestion instrument (Mars 6, CEM Corporation, USA). The Cd levels in the plant samples were determined using atomic absorbance spectrometry (ZEEnit^®^ 700P, Analytik Jena AG, Jena, Germany). The enrichment factor (EF) = Cd concentration in roots (shoots)/Cd concentration in soil, and the translocation factor (TF) = Cd concentration in shoots/Cd concentration in roots.

### 2.4. Soil Samples Analysis

To determine soil pH, 10 g of the air-dried soil sampl·es were weighed and mixed with distilled water. Soil pH was measured with a pH meter (STARTER2100, Ohaus, Parsippany, NJ, USA) [39]. To determine Cd concentration in soil, 0.15 g of dried soil samples were digested with 8 mL HNO_3_, 2 mL HF, and 1 mL H_2_O_2_, using a microwave digestion instrument (Mars 6, CEM Corporation, Matthews, NC, USA) [40]. To determine the Cd fractions in soil, Cd concentration in various soil fractions was extracted according to the modified BCR method [41]. In brief, 1.0 g dried soil samples were weighed. The exchangeable Cd form was extracted by 40 mL 0.11 mol·L^−1^ HAc on oscillator at room temperature for 16 h (250 r·min^−^^1^), the reducible Cd fraction was extracted by 0.5 mol·L^−1^ NH_2_OH·HCl on oscillator under the same conditions, and the oxidizable Cd fraction was digested with 30% H_2_O_2_ in 85 °C water. Then, 50 mL of 1 mol·L^−1^ NH_4_OAc was added after cooling and the residual Cd fraction was digested with 10 mL HNO_3_, 10 mL HF, and 3 mL HClO_4_ in a hot plate, dissolved with distilled water and transferred to a 25 mL volumetric flask. The Cd content and fractions in soil were determined using atomic absorbance spectrometry (ZEEnit^®^ 700P, Analytik Jena, AG, Jena, Germany).

### 2.5. Statistical Analysis

All data were expressed as the mean ± standard deviation of the three replicates. Statistical analysis was performed using SPSS 18.0 (IBM, New York, NY, USA), and data differences among treatments with the same material were analyzed by one-way ANOVA and the Duncan test (*p* < 0.05).

## 3. Results

### 3.1. Effects of Si on Plant Biomass

**Figure 1:**
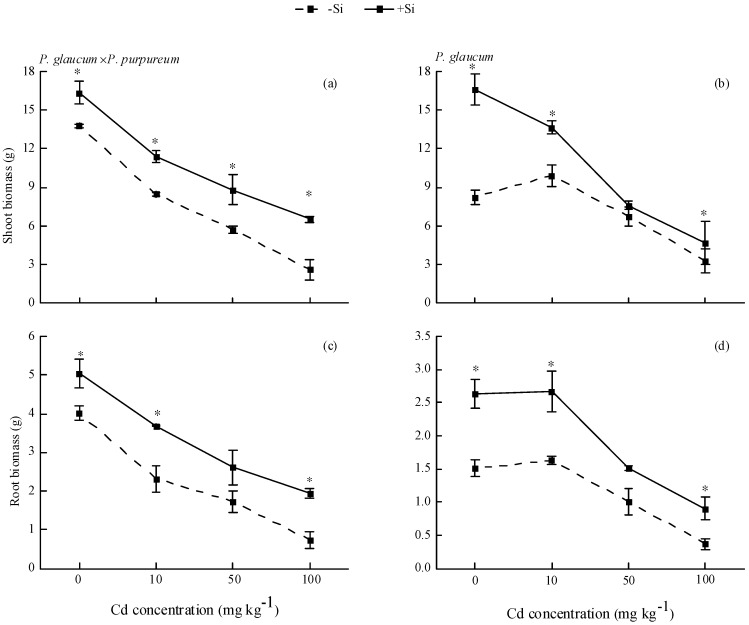
Effects of Si on the biomass of *Pennisetum* under Cd stress. (**a**,**c**) shoot and root biomass of *P. glaucum × P. purpureum*; (**b**,**d**) shoot and root biomass of *P. glaucum.* Values are mean ± SE (*n* = 3). * indicates significant difference between different treatments of the same variety, according to one-way ANOVA followed by the Duncan test (*p* < 0.05).

Si had beneficial effects in enhancing plant resistance against Cd stress for both *Pennisetum*. As Cd application level increased, the biomass in roots and shoots decreased gradually. For *P. glaucum × P. purpureum* (Figure 1a,c), Si addition significantly increased shoot biomass by 18.62–148.98% and root biomass by 25.94–164.68% under different Cd levels. Similarly, Si increased the shoot biomass by 12.65–101.46% and root biomass by 49.97–143.67% for *P. glaucum*, regardless of Cd stress (Figure 1b,d).

### 3.2. Effect of Si on Cd/Si Accumulation in Plants and the Enrichment Factor (EF)

A single Cd treatment did not influence plant Si concentration. Si application significantly increased Si uptake and reduced Cd concentration in roots and shoots for both varieties (Table 2). For *P. glaucum × P. purpureum*, Si addition increased Si concentration in shoots by 13.70–46.76% and in roots by 42.34–56.17%. Similarly, Si significantly increased Si concentration in shoots by 19.61–20.61% and in roots by 29.24–52.66% for *P. glaucum*, except for shoot Si concentration under the 0 and 100 mg·kg^−1^ Cd stress conditions (Table 2).

With the increase of the Cd treatment level, Cd concentration in roots and shoots increased significantly for both *Pennisetum* (Table 2). At 50 and 100 mg·kg^−1^ Cd levels, Cd concentration in roots of *P. glaucum × P. purpureum* were higher than those in the shoots. Similar results were obtained with *P. glaucum* under 100 mg·kg^−1^ Cd level. Si addition significantly reduced Cd concentration in the roots and shoots of the two varieties, except under the 10 mg·kg^−1^ Cd stress condition (Table 2). For *P. glaucum × P. purpureum*, Si application reduced Cd concentration by 23.38% and 54.26% in shoots and by 42.53% and 77.36% in roots under 50 and 100 mg·kg^−1^ Cd treatments, respectively. The corresponding reductions in *P. glaucum* were 21.53% and 36.71% in shoots and 52.72% and 43.82% in roots, respectively (Table 2). However, Si did not influence Cd accumulation in shoots and roots for both varieties under different Cd treatment levels.

The EF, which represents the degree of toxic metal element enrichment in plants, is the ratio of Cd content in each plant organ to the Cd content in soil. The TF, which represents the ability of toxic metal elements to transfer from root to shoot, is the ratio of the Cd content of the above-ground organs to the root. As shown in Table 2, for *P. glaucum × P. purpureum*, Si addition significantly decreased the EF in shoots by 50.00–67.18% and in roots by 66.67–74.42%, respectively, except for the EF in shoots under the 100 mg·kg^−1^ Cd treatment. In general, Si did not influence TF, except under 100 mg·kg^−1^ Cd treatment, where TF was 106.85% higher in Si-treated plants than in non-Si-treated ones. Similarly, for *P. glaucum*, EF values were decreased by 54.29% and 66.67% in shoots after silicon addition under 10 and 100 mg·kg^−1^ Cd treatments, respectively, and by 70.97% in roots under the 100 mg·kg^−1^ Cd. However, the TF decreased by 46.67% under 10 mg·kg^−1^ Cd and increased by 66.67% under 50 mg·kg^−1^ Cd after Si addition.

### 3.3. Effects of Si Application on Soil pH

**Figure 2:**
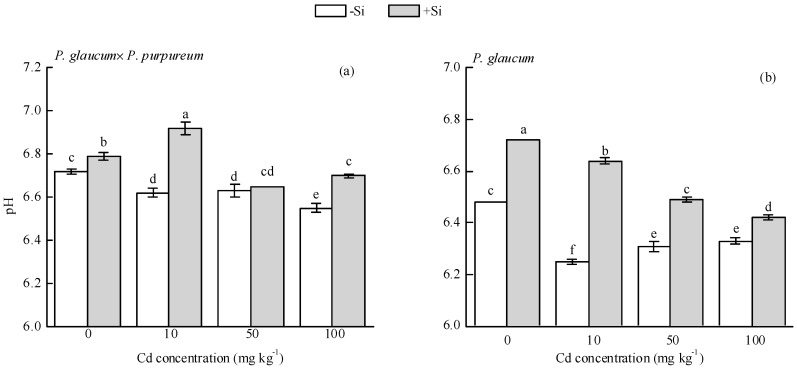
Effect of Si on soil pH of (**a**) *P. glaucum × P. purpureum* and (**b**) *P. glaucum*. Values are mean ± SE (*n* = 3). Values followed by the different letters indicate significant differences between different treatments of the same variety, according to one-way ANOVA and Duncan test (*p* < 0.05).

Si addition significantly increased soil pH (Figure 2). In *P. glaucum × P. purpureum*, the pH values of Si-treated soils were 0.07, 0.30, and 0.25 units higher than those of single Cd treatment (0, 10, and 100 mg·kg^−1^ Cd stress), respectively; similarly, in *P. glaucum*, the pH values of Si-treated soils were 0.24, 0.39, 0.02, and 0.09 units higher than those of single Cd treatment (0, 10, 50, and 100 mg·kg^−1^ Cd stress).

### 3.4. Effect of Si on Total Cd and Cd Fractions in Soil

**Figure 3:**
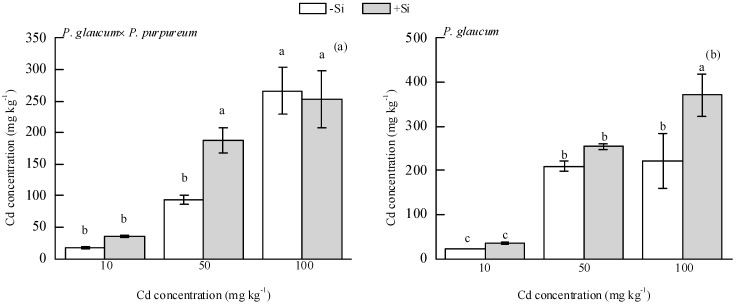
Effect of Si on soil total Cd concentration of (**a**) *P. glaucum × P. purpureum* and (**b**) *P. glaucum*. Values are mean ± SE (*n* = 3). Values followed by the different letters indicate significant differences between different treatments of the same variety, according to one-way ANOVA followed by the Duncan test (*p* < 0.05).

As shown in Figure 3, Si addition significantly increased total soil Cd concentration by 100.34% under the 50 mg·kg^−1^ Cd level for *P. glaucum × P. purpureum* (Figure 3a) and by 66.97% under the 100 mg·kg^−1^ Cd level for *P. glaucum* (Figure 3b).

**Figure 4:**
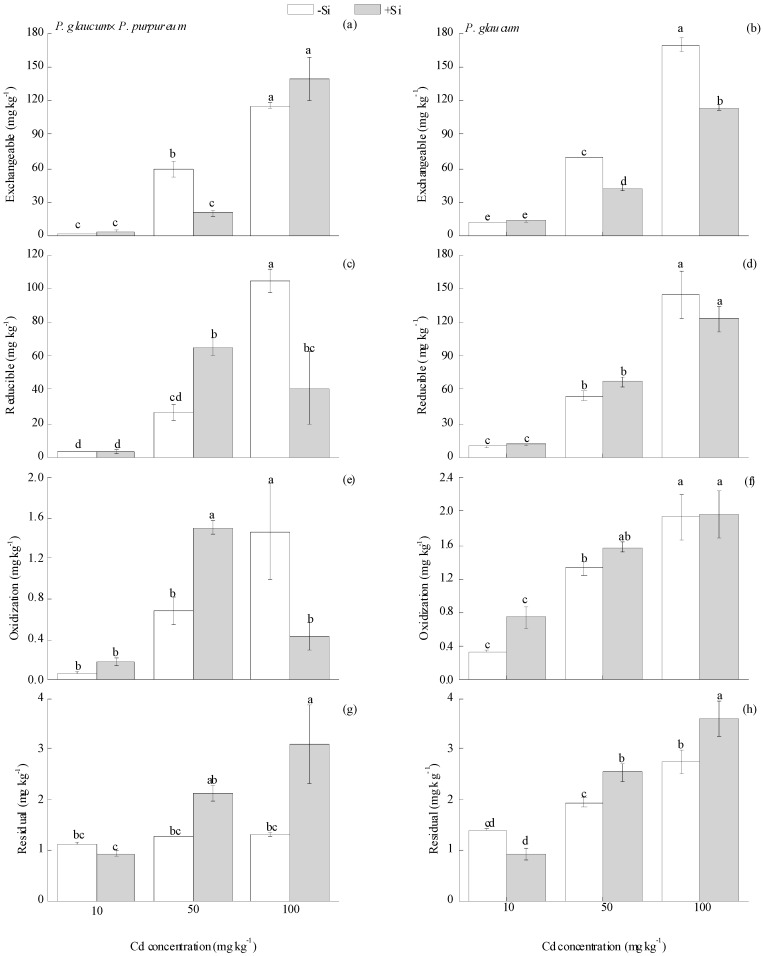
Effect of Si on the fractions of Cd in soil for (**a**,**c**,**e**,**g**) *P. glaucum × P. purpureum* and (**b**,**d**,**f**,**h**) *P. glaucum*. (**a**,**b**) Exchangeable Cd. (**c**,**d**) Reducible Cd. (**e**,**f**) Oxidizable Cd. (**g**,**h**) Residual Cd. Values followed by the different letters indicate significant differences between the different treatments of the same variety, according to one-way ANOVA followed by the Duncan test (*p* < 0.05).

Four fractions of Cd are tested in soil: Exchangeable, oxidizable, reducible, and residual fractions [42]. Si treatment had significant effects on the concentration of different fractions of soil Cd, but this effect varied among different Cd levels and *Pennisetum* varieties. Si did not influence soil Cd fractions under the 10 mg·kg^−1^ Cd level for both varieties. For *P. glaucum × P. purpureum* (Figure 4a,c,e,g), under the 50 mg·kg^−1^ Cd level, Si decreased soil exchangeable Cd by 65.90%, and increased reducible and oxidizable Cd by 145.22% and 120.26%, respectively. Under the 100 mg·kg^−1^ Cd level, Si decreased the reducible and oxidizable Cd by 60.79% and 70.43%, respectively, and increased the residual Cd by 136.07%. Similarly, Si had significant effects on soil exchangeable and residual Cd in *P. glaucum* (Figure 4b,d,f,h). For example, Si decreased exchangeable Cd by 39.28% and 32.96% and increased residual Cd by 31.38% and 30.97% under the 50 and 100 mg·kg^−1^ Cd levels, respectively.

## 4. Discussion

### 4.1. Si Mitigates Cd Toxicity and Improves Growth in Pennisetum

Cd causes numerous changes in physiological and biochemical functions and structural destruction, thereby leading to significant restriction of growth and development in plants [7,13,43]. Our study showed that Cd treatment significantly inhibited the growth of *Pennisetum*, and the increased Cd level caused an evident decrease in biomass (Figure 1). This outcome is similar to the results on *Pennisetum* by Zhang et al. [44]. However, exogenous Si application has a significant effect in enhancing the toxic metal element tolerance of plants and reducing metal toxicity [23,24,44,45,46,47]. Song et al. [48] found that the growth of Cd-tolerant cultivar and Cd-sensitive cultivar of two pakchoi (*Brassica chinensis* L.) was severely inhibited under Cd stress, but Si application mitigated the toxic effects. In this study, Si significantly increases root and shoot biomass and improves plant growth, regardless of Cd levels for both *Pennisetum* (Figure 1), thereby indicating the significant role of Si in alleviating Cd toxicity.

### 4.2. Si Impacts on Plant Cd Accumulation and Transportation

Plants can readily absorb Cd upon exposure through the roots and translocate the metal element to the above-ground parts. Our studies showed that Cd concentration in roots, stems, and leaves of the two varieties increased with the rise of the Cd treated level (Table 1). This outcome is consistent with the study in which Cd plant uptake increased proportionally to increasing Cd concentration [49]. However, Cd concentration in shoots was lower than those in roots for *P. glaucum × P. purpureum* under the 50 and 100 mg·kg^−1^ Cd levels and *P. glaucum* under 100 mg·kg^−1^ Cd. Therefore, *Pennisetum*, like many other plants, enriches toxic metal elements in roots [46,50].

The benefit of Si for plants under abiotic stress, including the reduced absorption and transportation of heavy metals by plants, is well known [51]. In our study, the Si concentration of roots and shoots in Si-treated plants was significantly higher (Table 2), and such upsurges were related to the increased plant resistance to Cd stress. Our study also found that Si application caused a significant decrease in Cd concentration in roots and shoots (Table 2) for both *Pennisetum* at the 50 and 100 mg·kg^−1^ Cd levels, while the Cd accumulation of plants did not change significantly (Table 2). Similar results were reported in other studies where Si addition significantly decreased Cd concentrations in both shoots and roots, regardless of Cd levels, but no significant effects on Cd accumulation were observed in shoots and roots [46,52]. As the amount of Cd accumulation is determined by both the Cd content and the biomass, Si-enhanced plant growth may induce the dilution effect, thereby leading to less toxicity [46,52]. Meanwhile, Cd concentration is reduced more in roots than in shoots with the addition of Si. This could be attributed to the reduction of Cd absorption. Furthermore, our study showed that Si application significantly reduced the EF values in roots and shoots of both *Pennisetum* (Table 2). Thus, plant Cd uptake was inhibited by Si and this is similar to the findings with wheat by Wu et al. [46]. The TF is used to evaluate the transferability of Cd from root to the shoot. In previous studies, Gao et al. [53] demonstrated that foliar spraying Si decreased Cd translocation from roots to stems and from stems to brown rice and increasing Cd translocation from stems to leaves in cultivars WYHZ. Shi et al. [54] found that Si deposition in the vicinity of endodermis could restrain the apoplastic transport of Cd and reduce the transport of Cd from roots to shoots. In our study, we found that Si had no consistent effects on TF for two varieties, reduced TF at 10 mg·kg^−1^ Cd level for *P. glaucum*, while having increased TF at 50 and 100 mg·kg^−1^ Cd level for *P. glaucum* × *P. purpureum* and *P. glaucum*, respectively, indicating variety difference of Cd translocation and other factors influencing the detoxification mechanism of Si on Cd toxicity. We speculated that Si reduces Cd toxicity mainly by reducing the uptake of Cd of *Pennisetum* rather than by reducing the transport of Cd. This conclusion is similar to the study of Wu [46] on wheat.

### 4.3. Silicon Influences Cd Bioavailability in Soil

Heavy metals with different fractions in soil show large differences in migration ability, phytoextractability, and bioavailability [55]. Exchangeable Cd has strong migration ability and can be directly absorbed by plants [56]. Reducible and oxidizable Cd can be activated under specific conditions, whereas residual Cd is highly stable in soil and is not considered to be bioavailable for organisms [57,58]. Previous studies have shown that Si has important impacts on the metal bioavailability in soil [27]. In this study, Si addition reduced exchangeable/oxidizable/reducible (Figure 4a,e,c, respectively) Cd in soil and increased residual Cd (Figure 4g) in *P. glaucum × P. purpureum* under the 50 and 100 mg·kg^−1^ Cd levels. Similarly, Si application resulted in the reduction of exchangeable Cd (Figure 4b) and the increase of residual Cd in soil (Figure 4h) in *P. glaucum*. Thus, Si addition caused the transformation of Cd fractions from availability into unavailability. Similar results were obtained in other investigations [23,26,59,60]. Liang et al. [23] reported that 400 mg·kg^−1^ Si application decreased water—and CaCl_2_—extractable fractions of Cd, but increased Fe–Mn oxide-bound fractions, therefore more Cd was found in the form of specific adsorbed or Fe–Mn oxide-bound fractions in Si-amended soil. Cunha et al. [59] found that silicon altered Cd and Zn fractions in soil and mitigated the toxicity of Cd in maize, reduced most bioavailable fractions, and increased the allocation of metals into highly stable fractions.

Soil pH plays an important role in the fractions of heavy metals. Moreover, it can control the solubility of heavy metal ions [61] and thus promote the transformation of heavy metals from bioavailable to residual fractions and reduce the toxicity of heavy metals [62]. A negative correlation is generally believed to exist between soil pH and heavy metal mobility and availability in soil [63]. In our study, Si addition significantly increased soil pH (Figure 2), which is similar to other studies [23,64]. Li et al. [65] reported that the addition of 800 mg Si kg^−1^ (Na_2_SiO_3_) to Pb-contaminated soil significantly increased soil pH, as well as the carbonate and residual bound fractions of Pb, but reduced the exchangeable fraction of Pb, consequently reducing plant availability of Pb. Ding et al. [66] found that rhizosphere soil pH increased with the increase of Si level under different Cr levels. This trend indicated that exogenous Si induced the alkalization of rhizosphere soil, promoted the formation of precipitation-bound and organic matter-bound Cr in Cr-contaminated soil, and probably improved the stability of Cr and decreased Cr uptake from Cr-contaminated soil. Combined with the above evidence, our study indicated that Si-mediated detoxification of Cd is related to the increase of soil pH and residual Cd fraction but reduction of available Cd fractions, thereby reducing the absorption of Cd by *Pennisetum*.

## 5. Conclusions

In summary, our results suggest that Si can significantly influence the behavior of Cd in soil by altering its availability and reducing its uptake by plants. Si addition to Cd-contaminated soil has an important role in in situ remediation by immobilizing Cd metal, thereby reducing Cd availability and improving plant growth. Additionally, the increases in soil pH and plant Si content resulting from Si application contribute to plant growth. Si impacts on *P. glaucum × P. purpureum* were more effective than those in *P. glaucum* in terms of reducing Cd uptake and increasing plant growth.

## Figures and Tables

**Table 1:** The basic soil chemical properties.

pH	Organic Matter (g·kg^−1^)	Total N (g·kg^−1^)	Total P (g·kg^−1^)	Total K (g·kg^−1^)	Available N (mg·kg^−1^)	Available P (mg·kg^−1^)	Available K (mg·kg^−1^)
5.58	111.32	1.50	0.21	11.66	155.34	57.30	174.10

**Table 2:** Cd enrichment of *Pennisetum* with/without Si.

Variety	Cd Treatments	Si Treatments	Si Concentration mg·kg^−^^1^		Cd Concentration mg·kg^−1^		Cd Accumulation mg·pot^−1^		Enrichment Factor (EF)		Translocation Factor (TF)
			Shoots	Roots	Shoots	Roots	Shoots	Roots	Shoots	Roots	
*P. glaucum* *× P. purpureum*	**0**	−Si	6.12 ± 0.14bcde	4.04 ± 0.46c							
		+Si	6.38 ± 0.20abc	6.29 ± 0.25b							
	10	−Si	5.53 ± 0e	4.7 ± 0.05c	22.79 ± 0.93d	15.78 ± 0.72cd	0.19 ± 0.01ab	0.04 ± 0a	1.31 ± 0.11a	0.90 ± 0.04a	1.45 ± 0.066a
		+Si	6.61 ± 0.29ab	7.34 ± 0.36a	15.38 ± 0.6d	10.7 ± 0.41d	0.18 ± 0.01b	0.04 ± 0a	0.43 ± 0.22b	0.30 ± 0.03c	1.44 ± 0.08a
	50	−Si	5.78 ± 0.11cde	4.38 ± 0.05c	44.95 ± 1.21b	34.47 ± 2.39b	0.26 ± 0.02ab	0.06 ± 0.01a	0.49 ± 0.04b	0.37 ± 0.03bc	1.31 ± 0.06a
		+Si	6.98 ± 0.38a	6.36 ± 0.24b	34.44 ± 1.08c	19.81 ± 1.48cd	0.31 ± 0.05a	0.05 ± 0.01a	0.19 ± 0.01c	0.11 ± 0.01d	1.76 ± 0.16a
	100	−Si	5.68 ± 0.09de	4.70 ± 0.05c	80.62 ± 3.95a	110.32 ± 7.29a	0.21 ± 0.07ab	0.08 ± 0.03a	0.32 ± 0.05bc	0.43 ± 0.07b	0.73 ± 0.02b
		+Si	6.18 ± 0.00bcd	6.69 ± 0.39ab	36.88 ± 5.29bc	24.98 ± 1.38bc	0.24 ± 0.03ab	0.05 ± 0a	0.16 ± 0.03c	0.11 ± 0.02d	1.51 ± 0.30a
*P. glaucum*	0	−Si	5.9 ± 0.15de	4.53 ± 0.34f							
		+Si	6.71 ± 0.06bc	6.79 ± 0.09cd							
	10	−Si	5.53 ± 0.00ef	6.19 ± 0.31d	16.05 ± 0.11d	9.87 ± 0.78d	0.16 ± 0.01a	0.02 ± 0c	0.70 ± 0.01a	0.43 ± 0.03ab	1.65 ± 0.13a
		+Si	8.11 ± 0.49a	8.00 ± 0.15ab	11.29 ± 0.98d	13.68 ± 2.77d	0.15 ± 0.01a	0.04 ± 0.01bc	0.32 ± 0.04b	0.39 ± 0.09ab	0.88 ± 0.14b
	50	−Si	6.6 ± 0.30bcd	5.91 ± 0.61de	34.88 ± 1.96b	68.88 ± 4.6b	0.23 ± 0.02a	0.07 ± 0.02a	0.17 ± 0.01c	0.33 ± 0.01bc	0.51 ± 0.03c
		+Si	7.29 ± 0.10b	8.67 ± 0.20a	27.37 ± 0.70c	32.57 ± 2.74c	0.21 ± 0a	0.05 ± 0ab	0.11 ± 0.00c	0.13 ± 0.01c	0.85 ± 0.08b
	100	−Si	4.98 ± 0.19f	4.88 ± 0.28ef	61.98 ± 2.52a	117.54 ± 6.15a	0.20 ± 0.05a	0.04 ± 0.01ab	0.33 ± 0.01b	0.62 ± 0.16a	0.53 ± 0.03c
		+Si	6.55 ± 0.05cd	7.45 ± 0.58bc	39.23 ± 3.75b	66.03 ± 5.77b	0.18 ± 0.06a	0.06 ± 0.01ab	0.11 ± 0.00c	0.18 ± 0.02bc	0.6 ± 0.06bc

Values are mean ± SE (*n* = 3). Values followed by the different letters indicate significant differences between different treatments of the same variety, according to one-way ANOVA followed by the Duncan test (*p* < 0.05).
